# Transcriptome-wide expression analysis of *MYB* gene family leads to functional characterization of flavonoid biosynthesis in fruit coloration of *Ziziphus* Mill

**DOI:** 10.3389/fpls.2023.1171288

**Published:** 2023-05-12

**Authors:** Noor Muhammad, Zhi Luo, Xin Zhao, Meng Yang, Zhiguo Liu, Mengjun Liu

**Affiliations:** ^1^ College of Horticulture, Hebei Agricultural University, Baoding, China; ^2^ Research Center of Chinese Jujube, College of Horticulture, Hebei Agricultural University, Baoding, China

**Keywords:** *Ziziphus*, MYB TFs, transcriptional regulation, flavonoid biosynthesis, transient expression, fruit coloration

## Abstract

The *Ziziphus mauritiana* Lam. and *Z. jujuba* Mill. are the two most economically important members of the genus *Ziziphus.* The fruit color of *Z. mauritiana* remains green throughout fruit development in the majority of commercial cultivars, whereas its close relative, *Z. jujuba* Mill. turns from green to red in all cultivars. However, the lack of transcriptomic and genomic information confines our understanding of the molecular mechanisms underlying fruit coloration in *Z. mauritiana* (Ber). In the present study, we performed the transcriptome-wide analysis of MYB transcription factors (TFs) genes in *Z. mauritiana* and *Z. jujuba*, and identified 56 ZmMYB and 60 ZjMYB TFs in *Z. mauritiana* and *Z. jujuba*, respectively. Through transcriptomic expression analysis, four similar *MYB* genes (*ZmMYB/ZjMYB13, ZmMYB/ZjMYB44, ZmMYB/ZjMYB50*, and *ZmMYB/ZjMYB56)* from *Z. mauritiana* and *Z. jujuba* were selected as candidate key genes regulating flavonoid biosynthesis. Among these genes, the *ZjMYB44* gene was transiently highly expressed in fruit, and flavonoid content accumulation also increased, indicating that this gene can influence flavonoid content during the period of fruit coloration in *Z. jujuba*. The current study adds to our understanding of the classification of genes, motif structure, and predicted functions of the MYB TFs, as well as identifying MYBs that regulate flavonoid biosynthesis in *Ziziphus* (*Z. mauritiana* and *Z. jujuba*). Based on this information, we concluded that *MYB44* is involved in the flavonoids biosynthesis pathway during the fruit coloring of *Ziziphus*. Our research results provide an important understanding of the molecular mechanism of flavonoid biosynthesis resulting in fruit coloration and laying a foundation for further genetic improvement of fruit color in *Ziziphus*.

## 1 Introduction

Transcription factors (TFs) are important regulators of gene expression in plants, directing a variety of physiological mechanisms such as growth, development, cell division, stress response, and fruit color development (Muthamilarasan et al., 2014; Zhang et al., 2018; Qing et al., 2019; Fan et al., 2020; Muhammad et al., 2022; Muhammad et al., 2022a; Muhammad et al., 2023). As a result, interpreting the molecular and physiological functions of these TFs is now a major research focus. Furthermore, TF research gained traction, and a slew of new studies had been published. In plants, MYB (myeloblastosis) transcription factors are ubiquitous in eukaryotic systems, and their function in plant flavonoids like anthocyanin biosynthesis was first discovered in maize (Paz-Ares et al., 1987).

After the initial identification of MYB in *Zea mays*, numerous MYB TFs genes were gradually discovered in a variety of different plants, including the *Arabidopsis thaliana* (Stracke et al., 2001). These MYBs are recognized by the family-specific function of a highly conserved MYB domain at the N-terminus (Yanhui et al., 2006; Stracke et al., 2007). Plant MYB proteins have an MYB DNA-binding domain that is extremely conserved, the MYB domain is a 50-53 amino acid segment or region that interacts with DNA in a sequence-specific fashion (Zhang et al., 2018; Qing et al., 2019). The MYB TFs are divided into four subfamilies depending on the number of highly conserved imperfect repeats in the DNA-binding domain: R3 MYB (MYB1R) has one repeat, R2R3 MYB has 2 repeats, R1R2R3 MYB (MYB3R) has 3 repeats, and 4R MYB has 4 repeats (Lin-Wang et al., 2010; Muhammad et al., 2022). Similarly, the R2R3-MYB is the biggest TF gene family in plants, with 126 genes discovered in *A. thaliana* (Stracke et al., 2001; Yanhui et al., 2006). In strawberries, the R2R3 MYB gene *FaMYB1* regulates anthocyanin and flavonol production (Aharoni et al., 2001; Lin-Wang et al., 2010).

According to numerous reports, MYB TFs are involved in physiological and metabolic pathways in plant species, as well as responding to various biotic and abiotic stresses (Qing et al., 2019). The role of these MYB TFs has already been studied in some plant species like *Physcomitrella patens* (Pu et al., 2020), potato (Li et al., 2020), *Casuarina equisetifolia* (Wang et al., 2021), *Dendrobium catenatum* (Zhang et al., 2021), *Hedychium coronarium* (Abbas et al., 2021), Woodland strawberry (Li et al., 2021), *Brassica napus* L (Li et al., 2021a), Sunflower (Li et al., 2020a), Pitaya (Xie et al., 2021), Pineapple (Liu et al., 2017), *Vaccinium corymbosum* (Wang et al., 2021a), Chinese jujube (Qing et al., 2019), and so on. Similarly, the role of MYB TFs has also been reported in color or pigment production in the plant species like Liriodendron (Yang et al., 2021), kiwi (Li et al., 2017), peach (Zhang et al., 2018), apple (Jiang et al., 2019), Chinese bayberry (Cao et al., 2021), *Pistacia chinensis* (Song et al., 2021), *Fragaria ananassa* (Liu et al., 2021a), *Populus deltoids* (Zhuang et al., 2021), and pepper (Liu et al., 2021). However, none of the comparative studies have been conducted in *Z. mauritiana* and *Z. jujuba* that describe the role of MYBs in flavonoid biosynthesis during fruit coloration.

The *Ziziphus* Mill. is a monetarily important genus of the Rhamnaceae family with roughly one hundred and seventy species found all over this planet (Liu et al., 2020). *Z. jujuba* and *Z. mauritiana* are the most economically important members of this genus. *Z. mauritiana* is native to the Indian and Pakistan Subcontinent (Muhammad et al., 2020; Muhammad et al., 2022b). Out of the 14 native *Ziziphus* species in China, the jujube is thought to have evolved straightforwardly from wild sour jujube, making it an excellent source for the introduction of a variety of useful attributes for the increase in jujube fruit quality (Liu et al., 2011; Zhao et al., 2014; Liu et al., 2020; Muhammad et al., 2022c). Jujube is also one of the oldest domesticated fruit trees, with findings of cultivation stretching back over 7,000 years (Uddin et al., 2021; Uddin et al., 2022a). Aside from being a major dry fruit crop, it is also a good resource of herbal remedies and a source of income for some 20 million Asian people (Liu and Wang, 2009; Liu et al., 2020; Uddin et al., 2021; Uddin et al., 2022a). Because of their high nutritive quality and assorted phytochemical composition, both species (*Z. jujuba* and *Z. mauritiana*) have become important cash crops (Wang et al., 2018; Muhammad et al., 2022c; Uddin et al., 2022). The primary bioactive elements of jujube fruits, which have been widely used as food, food additives, and flavoring, are vitamin C, triterpenoids, amino acids, carbohydrates, phenolics, flavonoids, or anthocyanin (Wu et al., 2012; Wang et al., 2018).

Our completion of the whole-genome sequence of *Z. jujuba* Mill. in 2014 (Liu et al., 2014) for the first time provided evidence for genome-wide profiling of the MYB superfamily. However, the lack of whole-genome sequencing of *Z. mauritiana* limits our insights in this context. MYB transcription factors play a role in many aspects of plant growth, development, metabolism, and fruit coloration. But the comparative study of MYB TFs in *Z. jujuba*, and *Z. mauritiana* have not yet been completely known and characterized, and the expression of *MYB* genes in different developmental stages of the fresh fruit of these two species is undefined. The motifs analysis and phylogenetic trees are all reported in this article. Furthermore, the transient overexpression of *ZjMYB44* significantly increased flavonoid accumulation in the *Z. jujuba* fruit. Similarly, the *ZjMYB44* gene was transiently highly expressed in fruit, and flavonoid content accumulation increased, indicating that this gene can influence flavonoid content leading to color regulation in *Ziziphus* fruit. Our findings can help characterize *ZjMYB* and *ZmMYB* genes, and this research will aid future further functional studies of *ZjMYB* and *ZmMYB* superfamily genes involved in the color development of *Z. jujuba* and *Z. mauritina* fruits.

## 2 Materials and methods

### 2.1 Identification of ZmMYB and ZjMYB protein sequences and database searches

The transcriptome data from *Z. mauritiana* and *Z. jujuba* was used to identify all of the presumed MYBs proteins. The Pfam database (http://pfam.xfam.org/) was searched to obtain the hidden Markov Model (HMM) profile for the MYB binding domain (Finn et al., 2016; Zhang et al., 2018). The presence of an MYB domain in the selected MYB proteins was further verified using the online program HMMER (https://www.ebi.ac.uk/Tools/hmmer/). The Arabidopsis Information Resource (TAIR) database was used to obtain Arabidopsis MYBs protein sequences and was then blasted using Blastp. The ZmMYB and ZjMYB proteins were identified using the Arabidopsis MYBs domain sequences as a reference. To evade repetition, multiple alignments were accomplished among the identified ZmMYB and ZjMYB protein sequences.

### 2.2 Sequence analysis and construction of the phylogenetic tree

The MEGA version 7.0 was used to create a phylogenetic tree employing the neighbor-joining approach (Tamura et al., 2013; Cui et al., 2018). Furthermore, the MYB protein sequences from Arabidopsis were obtained, and ClustalW was used to align Arabidopsis MYB protein sequences and the deduced amino-acid sequences of ZmMYBs and ZjMYBs (Chenna et al., 2003; Larkin et al., 2007; Cui et al., 2018). The phylogenetic tree between ZmMYBs and ZjMYBs and the known MYBs from Arabidopsis was then constructed using the neighbor-joining method and the MEGA7.0 program, and bootstrap analysis was performed with 1,000 replicates (Yang et al., 2015). The Compute *pI/Mw* online tool (http://web.expasy.org/compute pi/) was used to calculate the theoretical isoelectric point (*pI*) and molecular weight (*Mw*) of ZmMYB and ZjMYB proteins, and Cell-PLoc 2.0 (http://www.csbio.sjtu.edu.cn/bioinf/Cell-PLoc-2/) was used to predict their subcellular localization.

### 2.3 Conserved domain and motif analysis in MYB protein sequences

The MEME Suit 5.5.0 program (http://meme-suite.org/tools/meme) was used to recognize the motifs manifest in the MYBs proteins, and the conserved motifs of ZmMYB and ZjMYB proteins were identified (Bailey et al., 2009). The distribution of motifs was set to zero or one per sequence, the maximum number of motifs to be found was 10, and the default parameters are being used. Pfam (http://pfam.xfam.org) was used to define the conserved domains of ZmMYB and ZjMYB proteins. Thereafter, we used the TBTools software (https://github.com/CJ-Chen/TBtools) to visualize the Pfam, and MEME, results (Chen et al., 2020).

### 2.4 Treatment for plant materials (fruit)

The fruit samples of green-colored cultivars of Z*. mauritiana* (Gaolong) at different developmental stages were collected from Yunan Province, China. Fifteen cDNA libraries were generated using total RNA extracted from the *Z. mauritiana* green cultivars fruit peel and *Z. jujuba* red fruit cultivar (Jinsixiaozao) (three replicates per tissue; Junior fruit (JF: JF1, JF2, JF3), Before white riping fruit (BWRF: BWRF1, BWRF2, BWRF3); Half ripening fruit (HRF: HRF1, HRF2, HRF3); White-ripening fruit (WRF: WRF1, WRF2, WRF3), and Ripen fruit (RF: RF1, RF2, RF3). At the age of 30 (junior fruit, JF), 50 (before ripening fruit, BRF), 80 (white ripening fruit, WRF), 90 (half ripening fruit, HRF), 100 (full ripening fruit, RF) days after pollination (DAP), fruit specimens were harvested, and the samples were collected. The fruit skin samples (at least 10 fruits per sample) were promptly frozen in liquid nitrogen and preserved at -80°C for further investigation. Each tissue or fruit skin sample consisted of three biological replicates.

### 2.5 Transient expression of *ZjMYB13 ZjMYB44, ZjMYB50*, and *ZjMYB56* in *Z. jujuba* fruit

The red fruit cultivar was used at the white ripening stage in the transient expression assay. The *ZjMYB13, ZjMYB44, ZjMYB50*, and *ZjMYB56* were inserted into the pCambia1302 vector, and the recombinant plasmids (pCambia1302 vector) of *ZjMYB13, ZjMYB44, ZjMYB50*, and *ZjMYB56* were constructed and transferred to *Escherichia coli*, the recombinant plasmids *ZjMYB13*, *ZjMYB44*, *ZjMYB50*, and *ZjMYB56* were transformed into *Agrobacterium tumefaciens* GV3101. Fruits of ‘Dongzao’ jujube collected at the white ripe stage were injected with transformed *A. tumefaciens* containing *ZjMYB13*, *ZjMYB44*, *ZjMYB50*, and *ZjMYB56* respectively. Similarly, a pCambia1302 control was also set, and 20 ‘Dongzao’ fruits of the same (size, color value, hardness, etc.) and 1 mL of resuspended bacteria were injected along the shoulder, and one fruit was injected on two sides. The injection ended the dark treatment for 24h and was sampled after every 24h intervals, then after, fruits were ground and stored at -80˚C for subsequent flavonoids test and real-time quantitative assay.

### 2.6 Measurement of flavonoids

The total flavonoid content was determined by using the procedure as follows.

#### 2.6.1 Standard preparation of rutin solution

0.2 mg/ml of rutin solution was prepared. 0 mL, 1 mL, 2 mL, 3 mL, 4 mL, and 5 mL of rutin solution were taken and added to 0.6 mL of 5% sodium nitrite (NaNO_2_) solution. After allowing the solution to stand for 6 minutes mixed well, 0.4 mL 10% aluminum nitrate solution [10% Al(NO_3_)_3_] was added, and mixed properly, and after 6 min, 2 mL of 4% sodium hydroxide (NaOH) was added. The absolute ethanol was adjusted to 10 mL, then mixed, and after 30 min, the absorbance was measured using a UV-vis spectrophotometer at 511 nm.

#### 2.6.2 Sample determination

The sample material was mixed in 50 mL centrifuge tubes at 1:30 (g sample: mmL ethanol) and sonicated at 50°C for 40 min after 15 min centrifugation 1mL supernatant was removed. Poured 1 mL of sample (supernatant) into the tube, 0.6 mL of 5% sodium nitrite solution was added and was allowed to stand for 6 min. the 0.4 mL of 10% aluminum nitrate solution was added and mixed well. After 6 min 2 mL of 4% sodium hydroxide was added. The absolute ethanol was adjusted to 10 mL, then mixed, and after 30 min, the absorbance was measured using a UV-vis spectrophotometer at 511 nm.

### 2.7 Real-time quantitative detection

RNA was extracted from fruit peel at five developmental stages of fruit. Real-time quantitative primers were designed with the NCBI website. Three pairs were designed to screen the best primers and were used for subsequent real-time quantitative detection as shown in **Table S1**. qRT-PCR analysis was performed on a Bio-Rad real-time quantitative instrument. A real-time quantification kit (Tiangen Biotechnology, China) was used. The 20 µL reaction system contained 10 µL of 2 SYBR Premix, 10 µM of primers 0.4 µL each, 1 µL of diluted cDNA, and 8.2 µL ddH_2_O. They were incubated at 94°C for 15 min, followed by 15 s at 94°C, 55 to 63°C, and 15 s at 72°C. The 2^−ΔΔCT^ method (Livak and Schmittgen, 2001) was used to analyze gene expression levels, and *ZjActin* was used as an internal control (Zhang et al., 2015; Shi et al., 2020; Zhang et al., 2020). All qRT-PCR primers are presented the **Table S1**.

## 3 Results

### 3.1 Phylogenetic analyses and function predictions of ZmMYBs and ZjMYBs

In the present study, we performed the transcriptome-wide analysis of MYB TFs genes in *Ziziphus* and identified 56 ZmMYB (ZmMYB1 through ZmMYB56) and 60 ZjMYB (ZjMYB1 through ZjMYB60) transcription factors (TFs) in *Z. mauritiana* and *Z. jujuba*, respectively. To assess the evolutionary pattern among *Ziziphus* species (*Z. mauritiana* and *Z. jujuba*) and Arabidopsis MYB proteins, the deduced amino acid sequences of the MYB proteins identified from Arabidopsis and *Ziziphus* transcriptomes were completely aligned. After that, a combined phylogenetic tree was constructed using the neighbor-joining method and bootstrap analysis (1,000 reiterations) (**Figure S1**).

**Figure 1:**
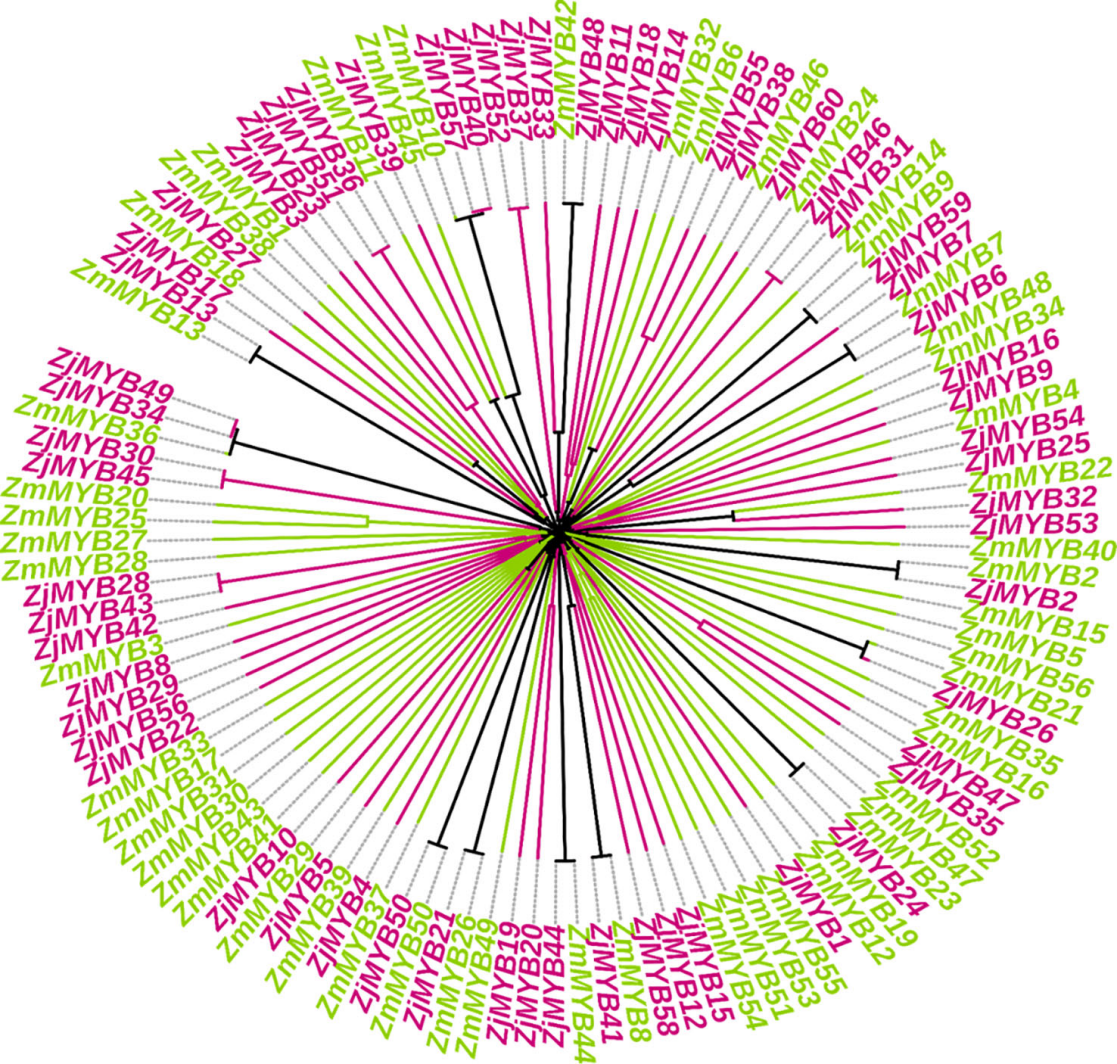
Comparative phylogenetic analysis of *Z. mauritiana* and *Z. jujuba* MYB proteins and are labeled by two different colors. The ZmMYB and ZjMYB represent *Z.mauritiana* and *Z. jujuba* MYB proteins respectively. ZmMYBs are in yellow-green and ZjMYBs are represented in red-violet color.

By aligning the entire set of predicted MYB protein sequences from *A. thaliana* (171), Chinese jujube (60), and *Z. mauritiana* (56), a phylogenetic tree of AtMYB, ZjMYB, ZmMYBs protein was constructed (**Figure S1**). Furthermore, in a combined phylogenetic tree of *Z. jujuba* and *Z. mauritiana*, 13 pairs of putative orthologous proteins (ZmMYB12 and ZjMYB13, ZmMYB11 and ZjMYB39, ZmMYB42 and ZjMYB48, ZmMYB9 and ZjMYB9, ZmMYB7 and ZjMYB6, ZmMYB22 and ZjMYB32, ZmMYB2 and ZjMYB2, ZmMYB21 and ZjMYB26, ZmMYB23 and ZjMYB24, ZmMYB8 and ZjMYB41, ZmMYB44 and ZjMYB44, ZmMYB26 and ZjMYB21, ZmMYB50 and ZjMYB50) were identified. In contrast, 2 pairs of paralogous MYB family proteins were identified in *Z. mauritiana*: ZmMYB53 and ZmMYB51, ZmMYB25 and ZmMYB20. Similarly, 10 pairs of paralogous MYB family proteins were identified in *Z. jujuba*, i.e., ZjMYB51 and ZjMYB36, ZjMYB57 and ZjMYB40, ZjMYB52 and ZjMYB37, ZjMYB55 and ZjMYB38, ZjMYB46 and ZjMYB31, ZjMYB47 and ZjMYB35, ZjMYB20 and ZjMYB19, ZjMYB43 and ZjMYB28, ZjMYB45 and ZjMYB30, ZjMYB35 and ZjMYB49 as shown in **Figure 1**.

### 3.2 Conserved sequences in *Ziziphus* MYB proteins

**Figure 2:**
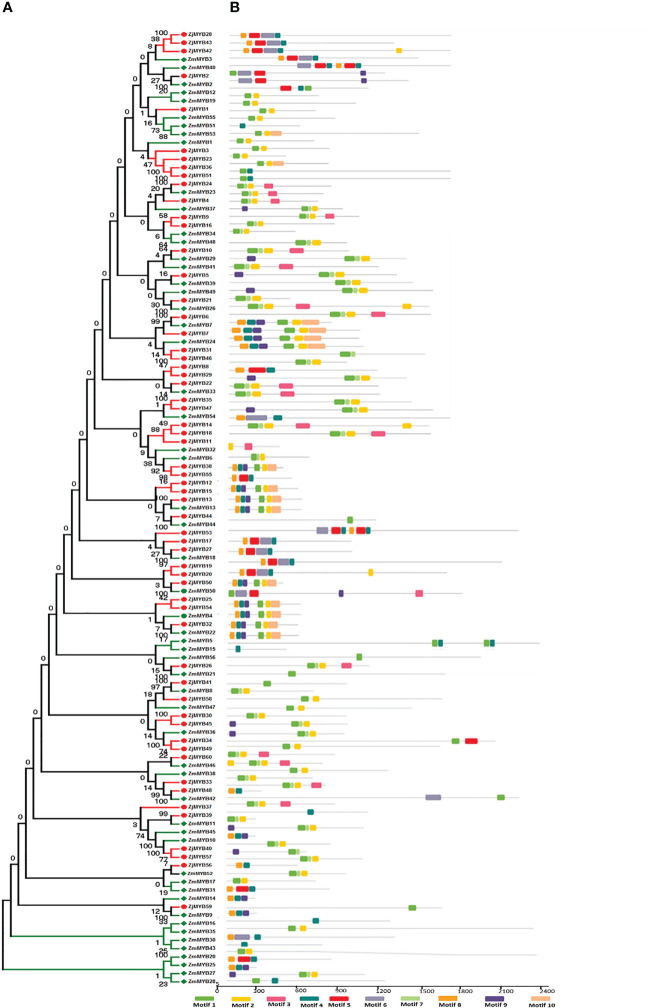
Phylogenetic relationships, the architecture of conserved protein motifs in R2R3-MYB proteins from *Z. mauritiana* and *Z. jujuba*. **(A)** The phylogenetic tree was constructed with 56 and 60 R2R3-MYB proteins from *Z. mauritiana* and *Z. jujuba*, respectively. The ZjMYBs are represented by red color while ZmMYBs are represented by green color. **(B)** Architecture of conserved protein motifs in different subfamilies. The colored boxes designated the different motifs as listed at the bottom of the figure.

**Figure 3:**
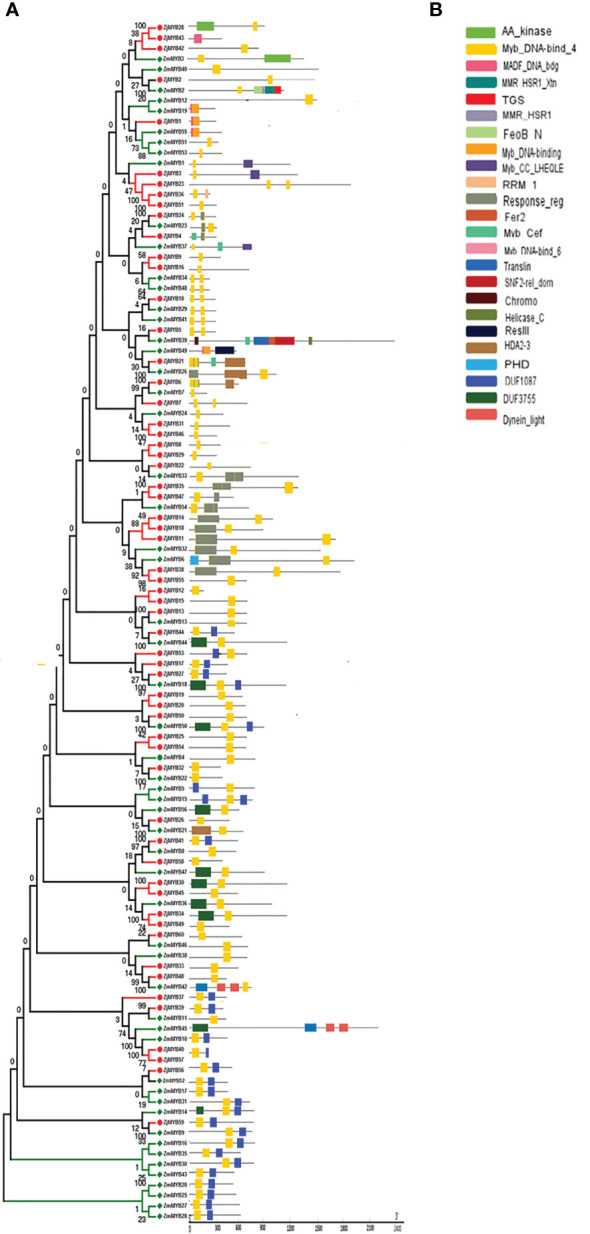
Phylogenetic relationships, the architecture of conserved MYBs domains in R2R3-MYB proteins from *Z. mauritiana* and *Z. jujuba*. **(A)** The phylogenetic tree was constructed with 56 and 60 R2R3-MYB proteins from *Z. mauritiana* and *Z. jujuba* respectively. **(B)** The architecture of conserved MYBs domains in different subfamilies. The colored boxes designated the different MYBs domains as shown in the figure.

The MEME and TBtool were used to find the conserved motifs. The findings demonstrated that the majority of R2R3-MYB proteins belonging to the same subfamily shared similar motifs, further demonstrating how closely related these proteins’ evolutionary relationships are to those of the phylogenetic tree (**Figure 2**). MYB TFs contain the MYB DNA-binding domain, which is usually found near the protein’s N-terminus. Based on MEME analysis, and hidden Markov Model (HMM) profile for the MYB binding domain, the MYB domains were found in all of the deduced MYB protein sequences in *Ziziphus* (*Z. mauritiana* and *Z. jujuba*), and each domain sequence contained 50-53 amino acid residues (**Figure 3**). Further, the sequence logos showed the presence of conserved sequences at specific positions. A to J depicted the repeats of *Z. mauritiana* MYB protein sequences, and M to V presented *Z. jujuba* MYB protein sequences. These findings indicated that the majority of amino acids are conservatively represented in each repeat of two *Ziziphus* species (**Figure S2**).

Furthermore, the MYB domain is the core motif of MYB transcription factors, and it is intimately associated with binding to the promoters of their target genes. Multiple sequence alignment analyses of 116 MYBs from *Ziziphus* (56 from *Z. mauritiana* and 60 from *Z. jujuba*) were used to generate sequence logos to investigate conservation at specific positions in the MYB domain. The R2 and R3 repeats contain many conserved amino acids, including the distinctive Trp (W) residues that are regarded landmarks of the MYB domain, as shown in **Figure S2**. The R2 repeat contains three conserved Trp residues as sown in **Figure S2**.

### 3.3 Subcellular localization prediction analysis

The 60 *Z. jujuba* and 56 *Z. mauritiana* isolated MYB protein sequences from the transcriptome were designated as ZjMYB1 through ZjMYB60 and ZmMYB1 through ZmMYB56, respectively. The 60 ZjMYB and 56 ZmMYB proteins were all predicted to be localized in the nucleus as shown in **Table 1**.

**Table 1:** Prediction of subcellular localization of MYB proteins through Cell-PLoc 2.0.

Gene	*pI*	*Mw* (Da)	Subcellular localization	Gene	*pI*	*Mw* (Da)	Subcellular localization
*Ziziphus jujuba*				*Z. mauritiana*			
*ZjMYB1*	5.6	116390.3	Nuclear	*ZmMYB1*	8.58	138572.9	Nuclear
*ZjMYB2*	6.75	64872.75	Nuclear	*ZmMYB2*	6.69	57289.19	Nuclear
*ZjMYB3*	5.4	103638.8	Nuclear	*ZmMYB3*	9.72	59500.99	Nuclear
*ZjMYB4*	8.84	75568.77	Nuclear	*ZmMYB4*	5.9	127347.4	Nuclear
*ZjMYB5*	9.26	123766.2	Nuclear	*ZmMYB5*	5.42	103072.1	Nuclear
*ZjMYB6*	9.28	63143.98	Nuclear	*ZmMYB6*	6.6	26384.45	Nuclear
*ZjMYB7*	8.29	72449.58	Nuclear	*ZmMYB7*	5.35	111835.2	Nuclear
*ZjMYB8*	9.09	56245.43	Nuclear	*ZmMYB8*	9.21	34120.11	Nuclear
*ZjMYB9*	9.02	34881.18	Nuclear	*ZmMYB9*	8.98	35018.44	Nuclear
*ZjMYB10*	6.87	49955.95	Nuclear	*ZmMYB10*	6.64	31436.87	Nuclear
*ZjMYB11*	9.79	33456.57	Nuclear	*ZmMYB11*	7.05	32374.26	Nuclear
*ZjMYB12*	6.61	41906.98	Nuclear	*ZmMYB12*	6.2	199098.9	Nuclear
*ZjMYB13*	8.16	72375.08	Nuclear	*ZmMYB13*	5.76	44385.53	Nuclear
*ZjMYB14*	8.9	75829.62	Nuclear	*ZmMYB14*	8.03	118632.1	Nuclear
*ZjMYB15*	8.53	33345.95	Nuclear	*ZmMYB15*	7.65	50187.87	Nuclear
*ZjMYB16*	8.87	38752.64	Nuclear	*ZmMYB16*	5.8	72078.03	Nuclear
*ZjMYB17*	9.61	22611.91	Nuclear	*ZmMYB17*	5.89	41295.71	Nuclear
*ZjMYB18*	7.73	38204.93	Nuclear	*ZmMYB18*	6.01	31016.8	Nuclear
*ZjMYB19*	9.04	136044.2	Nuclear	*ZmMYB19*	6.22	73712.99	Nuclear
*ZjMYB20*	8.98	127842.5	Nuclear	*ZmMYB20*	5.58	66782.53	Nuclear
*ZjMYB21*	8.55	40101.51	Nuclear	*ZmMYB21*	8.39	42202.53	Nuclear
*ZjMYB22*	6.43	36630.78	Nuclear	*ZmMYB22*	7.21	41854.28	Nuclear
*ZjMYB23*	6.35	34846.39	Nuclear	*ZmMYB23*	6.43	45212.61	Nuclear
*ZjMYB24*	6.43	45141.53	Nuclear	*ZmMYB24*	8.6	96585.31	Nuclear
*ZjMYB25*	5.18	51108.22	Nuclear	*ZmMYB25*	5.89	76208.73	Nuclear
*ZjMYB26*	8.38	42195.62	Nuclear	*ZmMYB26*	8.55	40101.51	Nuclear
*ZjMYB27*	6.46	26893.04	Nuclear	*ZmMYB27*	8.81	33263.27	Nuclear
*ZjMYB28*	9.74	29736.33	Nuclear	*ZmMYB28*	4.63	59524.86	Nuclear
*ZjMYB29*	7.86	29554.97	Nuclear	*ZmMYB29*	8.92	31921.08	Nuclear
*ZjMYB30*	5.87	44338.48	Nuclear	*ZmMYB30*	6.1	38280.65	Nuclear
*ZjMYB31*	7.18	38027.64	Nuclear	*ZmMYB31*	4.16	12866.33	Nuclear
*ZjMYB32*	7.23	41832.24	Nuclear	*ZmMYB32*	5.3	106604.1	Nuclear
*ZjMYB33*	8.68	46866.62	Nuclear	*ZmMYB33*	8.94	40620.42	Nuclear
*ZjMYB34*	8.11	49997.36	Nuclear	*ZmMYB34*	4.28	53787.95	Nuclear
*ZjMYB35*	9.42	15711.76	Nuclear	*ZmMYB35*	6.05	11224.54	Nuclear
*ZjMYB36*	6.82	50324.06	Nuclear	*ZmMYB36*	8.11	50031.38	Nuclear
*ZjMYB37*	5.65	50507.13	Nuclear	*ZmMYB37*	8.93	11494.79	Nuclear
*ZjMYB38*	6.6	26384.45	Nuclear	*ZmMYB38*	9.02	38175.4	Nuclear
*ZjMYB39*	7.05	32374.26	Nuclear	*ZmMYB39*	5.41	30988.75	Nuclear
*ZjMYB40*	6.64	31406.8	Nuclear	*ZmMYB40*	8.65	52166.68	Nuclear
*ZjMYB41*	9.21	34120.11	Nuclear	*ZmMYB41*	7.81	26959.36	Nuclear
*ZjMYB42*	6.05	49602.36	Nuclear	*ZmMYB42*	8.68	47138.88	Nuclear
*ZjMYB43*	9.74	29736.33	Nuclear	*ZmMYB43*	5.89	33354.26	Nuclear
*ZjMYB44*	7.86	29554.97	Nuclear	*ZmMYB44*	8.52	79170.26	Nuclear
*ZjMYB45*	5.87	44338.48	Nuclear	*ZmMYB45*	9.19	10886.16	Nuclear
*ZjMYB46*	7.18	38027.64	Nuclear	*ZmMYB46*	8.66	75647.83	Nuclear
*ZjMYB47*	7.18	38027.64	Nuclear	*ZmMYB47*	9.47	11566.09	Nuclear
*ZjMYB48*	8.68	46866.62	Nuclear	*ZmMYB48*	4.39	58702.69	Nuclear
*ZjMYB49*	8.11	49997.36	Nuclear	*ZmMYB49*	9.22	148117.2	Nuclear
*ZjMYB50*	9.42	15711.76	Nuclear	*ZmMYB50*	6.05	110860.8	Nuclear
*ZjMYB51*	6.82	50324.06	Nuclear	*ZmMYB51*	5.79	34936.19	Nuclear
*ZjMYB52*	5.65	50507.13	Nuclear	*ZmMYB52*	7.52	256800.6	Nuclear
*ZjMYB53*	7.71	24529.58	Nuclear	*ZmMYB53*	9.76	41846.95	Nuclear
*ZjMYB54*	6.77	38645.42	Nuclear	*ZmMYB54*	9.66	40911.17	Nuclear
*ZjMYB55*	6.6	26384.45	Nuclear	*ZmMYB55*	5.81	34015.09	Nuclear
*ZjMYB56*	7.05	32374.26	Nuclear	*ZmMYB56*	6.4	57914.89	Nuclear
*ZjMYB57*	6.64	31406.8	Nuclear				
*ZjMYB58*	9.21	34120.11	Nuclear				
*ZjMYB59*	9.1	35040.49	Nuclear				
*ZjMYB60*	6.73	68806.91	Nuclear				

### 3.4 Expression patterns of *MYB* genes from transcriptome of *Z. mauritiana* and *Z. jujuba* involved in flavonoid biosynthesis/fruit coloration

**Figure 4:**
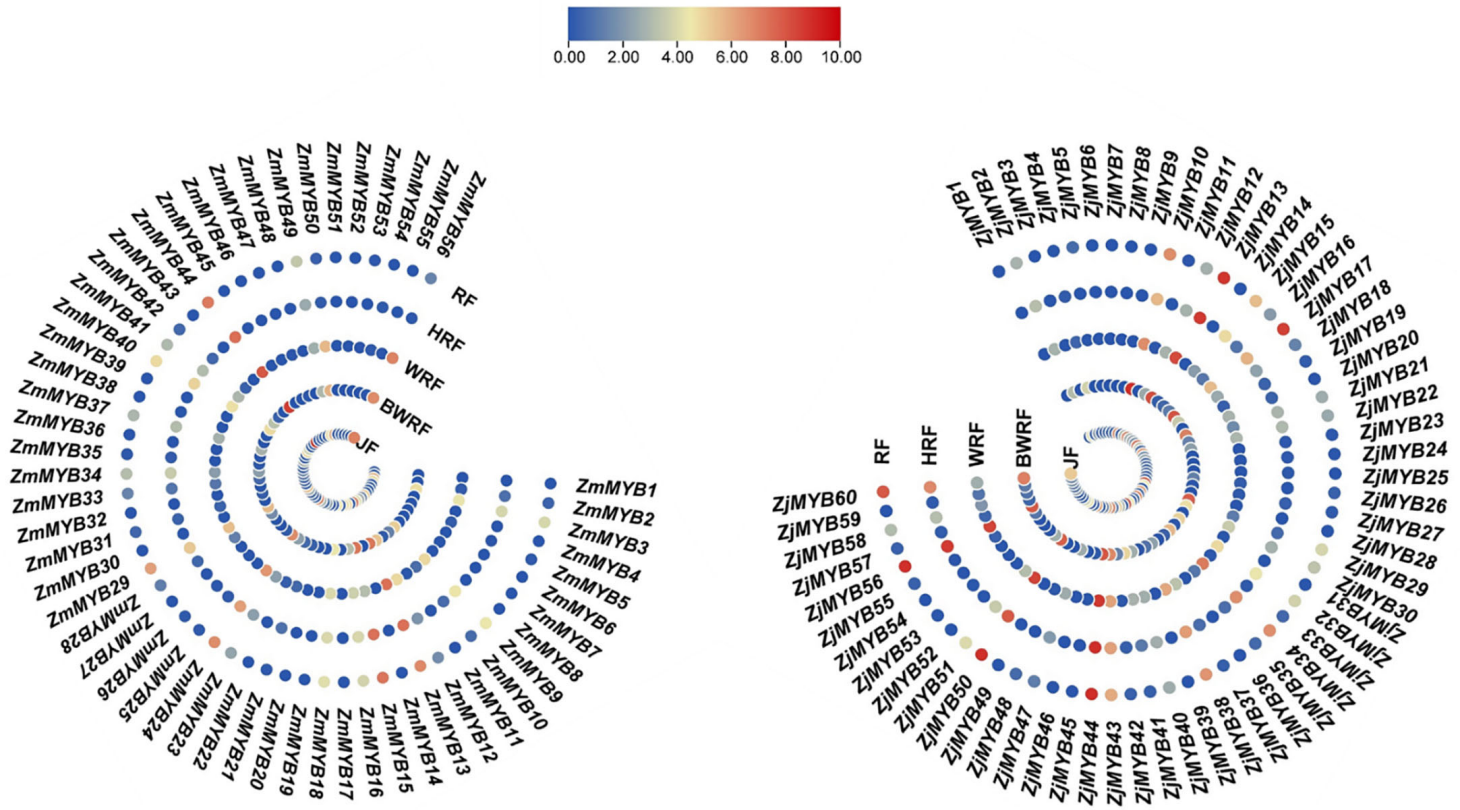
Differential expression of *ZmMYB* and *ZjMYB* genes in different developmental stages of *Ziziphus mauritiana* and *Z. jujuba* fruit. The Non-significance is blue and the Significant is shown as red in color. The *ZmMYB* gene that has been up-regulated is red, while the down-regulated is shown as blue in this figure. JF, BWRF, WRF, HRF, and RF represent junior fruit, before white ripening, white ripening, half ripening, and ripen fruit developmental stages, respectively.

The concentration levels of plant flavonoid pigments play a significant role in fruit color change. Our present transcriptomic study of MYB unveiled the important potential *ZmMYB*/*ZjMYB* genes, i.e., *ZmMYB*/*ZjMYB13*, *ZmMYB*/*ZjMYB44*, *ZmMYB*/*ZjMYB50*, and *ZmMYB*/*ZjMYB56*, involved in the flavonoid biosynthesis during the fruit developmental stages or during the period of fruit color change in *Z. mauritiana* (green color fruit) and *Z. jujuba* (red color fruit). The four important potential *MYB* genes (*ZmMYB*/*ZjMYB13*, *ZmMYB*/*ZjMYB44*, *ZmMYB*/*ZjMYB50*, and *ZmMYB*/*ZjMYB56*) were dramatically down-regulated or with low expression in *Z. mauritiana*, which is probably concomitant with the lack of flavonoid color pigments content in green fruit cultivars of *Z. mauritiana*. The *ZmMYB44* was significantly downregulated in fruit developmental stages of *Z. mauritiana* while this gene (*ZjMYB44*) was upregulated in fruit developmental stages of *Z. jujuba* as shown in **Figure 4**. Moreover, in *Z. mauritiana*, the expression level of this gene was highest in the junior fruit (JF) developmental stage (**Figure 4**), whereas in *Z. jujuba*, the high expression level was observed in the full ripening fruit (RF) stage (**Figure 4**). Similarly, the high expression of *ZmMYB56* was found in JF (Junior fruit) and BWRF (Before white ripening fruit) stages and then decreased the expression in WRF (White ripening fruit), HRF (Half ripening fruit), and RF (Ripen fruit) stages of fruit development in *Z. mauritiana*. Further, the expression of *ZmMYB*/*ZjMYB13* and *ZmMYB*/*ZjMYB50* genes were downregulated in *Z. mauritiana* fruit developmental stages and was upregulated in *Z. jujuba* fruit as shown in **Figure 4**. The rest of the genes showed no significant expression in the fruit developmental stages of both *Z. mauritiana* and *Z. jujuba*. Thus these four *ZmMYB/ZjMYB* genes of *Z. mauritiana* and *Z. jujuba* may be the possible key genes for the fruit color change in *Ziziphus* Mill.

### 3.5 qRT-PCR expression analysis

**Figure 5:**
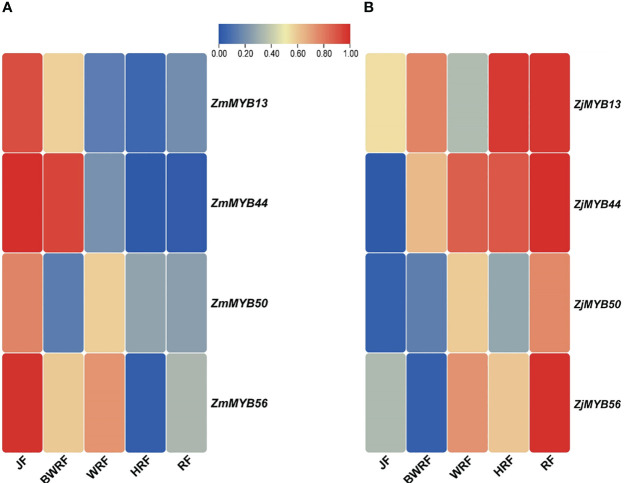
Validation of differential expression of the *MYB* genes (*ZmMYB13, ZmMYB44, ZmMYB50*, and *ZmMYB56* and *ZjMYB13*, *ZjMYB44*, *ZjMYB50*, and *ZjMYB56*) among five stages of fruit development of *Z. mauritiana* and *Z. jujuba* using qRT-PCR. The red color represents the high expression of the *MYB* genes and the blue color represents the low-level expression in corresponding fruit developmental stages. The **(A)** represents the expression level of *MYB* (*ZmMYB13*, *ZmMYB44*, *ZmMYB50*, and *ZmMYB56*) genes in *Z. mauritiana* while **(B)** represents the expression level of the same *MYB* (*ZjMYB13*, *ZjMYB44*, *ZjMYB50*, and *ZjMYB56*) genes in *Z. jujuba*. The JF stands for (Junior fruit), BWRF stands for (Before white ripening stage), WRF represents (White ripening stage), HRF stands for (Half ripening stage), and RF represents (ripen stage) of fruit development.

To validate the possible role of the 4 key *MYB* genes obtained from transcriptome data of *Z. mauritiana* and *Z. jujuba*, we conducted the qRT-PCR expression analysis. The expression of *ZmMYB13* was high at the junior fruit developmental stage of *Z. mauritania* but gradually decreased its expression at BWRF (before white ripening), WRF (white ripening), HRF (half ripening), RF (ripe fruit) stages of fruit development. While the expression of this gene (*ZjMYB13*) was high and upregulated in the fruit developmental stages of *Z. jujuba* except for WRF (white ripening) stage. Similarly, the expression of *ZmMYB44* was significantly downregulated in the fruit developmental stages of *Z. mauritiana* while the same gene *ZjMYB44* was significantly upregulated in the fruit developmental stages of *Z. jujuba*, confirming its expression level presented by the transcriptomic data. Similarly, *ZmMYB*/*ZjMYB56* presented downregulated trend in *Z. mauritiana* and upregulated trend in *Z. jujuba*. Moreover, the expression pattern of *ZmMYB*/*ZjMYB50* was not significant at all as shown in **Figure 5**.

### 3.6 Transient expression assay and flavonoid determination

**Figure 6:**
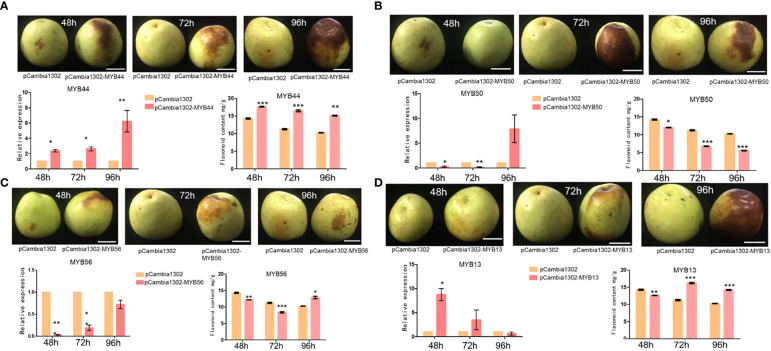
The transient expression of 4 *MYB* genes (*ZjMYB13*, *ZjMYB44*, *ZjMYB50*, and *ZjMYB56*) in jujube fruit. pCambia1302 was used as an empty vector. **(A)** The phenotype of pCambia1302, pCambia1302-MYB44, expression and flavonoid content of pCambia1302, pCambia1302-MYB44 of *Z. jujuba* fruits. **(B)** The phenotype of pCambia1302, pCambia1302-MYB50, expression and flavonoid content of pCambia1302, pCambia1302 MYB50 of *Z. jujuba* fruits. **(C)** The phenotype of pCambia-1302, pCambia1302-MYB56, expression and flavonoid content of pCambia-1302, pCambia1302-MYB56 of *Z. jujuba* fruits. **(D)** The phenotype of pCambia-1302, pCambia1302-MYB13, relative expression level and flavonoid content of pCambia1302, pCambia1302-MYB13 of *Z. jujuba* fruits.

To further validate the transcriptome and qRT-PCR-based expression analysis results of the selected *MYB* genes (*ZmMYB13, ZmMYB44, ZmMYB50*, and *ZmMYB56* and *ZjMYB13*, *ZjMYB44*, *ZjMYB50*, and *ZjMYB56*), we performed *in vivo* transient Agrobacterium infiltration assays on jujube fruit. We injected the plasmids ZjMYB44-pCambia1302, ZjMYB50-pCambia1302, ZjMYB56-pCambia1302, and ZjMYB13- pCambia1302 into the pericarps of jujube fruits. Compare to the control, the high transient expression of *ZjMYB44* significantly increased flavonoid accumulation in the fruit around the injection site (**Figure 6A**). Flavonoid biosynthetic *ZjMYB44* gene was consistently up-regulated in the fruit near the injection site. Thus, high transient expression of the *ZjMYB44* gene in fruit increased flavonoid accumulation, indicating that this gene is a key influencer of flavonoid content during the period of fruit color change in *Z. jujuba* as shown in **Figure 6A**. On the other hand, *ZjMYB50* gene was transiently expressed in fruit only at 96h after the injection with a constant decrease of flavonoid content, indicating that this gene cannot improve the flavonoid content accumulation (**Figure 6B**). Furthermore, *ZjMYB56* was transiently expressed in fruit, but the gene was not highly expressed in fruit and the flavonoid content was decreased, thus indicating that this gene cannot affect the flavonoid accumulation in the fruit peel (**Figure 6C**). Moreover, the *ZjMYB13* gene was transiently expressed in the fruit, the expression of *ZjMYB13* gene was high in the fruit, and the flavonoid content first decreased and then rose after 72h, demonstrating that this gene might indirectly induce the flavonoid content in the fruit peel of jujube (**Figure 6D**).

## 4 Discussion

The MYB TFs are engaged in physiological and biochemical mechanisms in plants, as well as responses to abiotic and biotic stresses (Raffaele et al., 2008; Qing et al., 2019), and the function of MYB has been extensively studied. MYB TFs are also engaged in flavonoid biosynthesis. For example, overexpression of the Arabidopsis *AtMYB12* gene increased flavonoid content accumulation under low temperatures in a light-dependent fashion (Bhatia et al., 2018). The *MYB10* gene in *Fragaria ananassa* plays a general regulatory role in the flavonoid/phenylpropanoid pathway during fruit ripening (Medina-Puche et al., 2014; Qing et al., 2019).

The MYB is the largest TF gene family in plants that is divided into four subfamilies based on the number of MYB imperfect tandem repeats (Rs) in the proteins: R3 MYB (MYB1R) has one repeat, R2R3 MYB has 2 repeats, R1R2R3 MYB (MYB3R) has 3 repeats, and 4R MYB has 4 repeats (Lin-Wang et al., 2010). Only R2R3-MYB plays a role in flavonoid or anthocyanin biosynthesis, with 126 genes discovered in *A. thaliana* (Stracke et al., 2001; Yanhui et al., 2006). In a genome-wide characterization and expression analysis of the MYB superfamily genes in jujube Qing et al. (2019) discovered 99 R2R3-MYB genes and 171 MYB genes in total. Due to the lack of genomic information on *Z. mauritiana*, we used transcriptome-wide analysis of the fruit peel and identified 60 MYBs in jujube (*Z. jujuba*) and 56 MYBs in Ber (*Z. msuritiana*). The number of MYBs decreased in jujube, this may be due to the fact that the genome contains all of the genes in a cell’s DNA (Hamilton and Robin Buell, 2012; Chen et al., 2019), whereas the transcriptome only contains those that are expressed within a specific tissue of the plant species (Ward et al., 2012).

The evolution of common ancestors for plant species and subsequent biological evolution research showed that orthologous genes could have contributed to the evolutionary developments of inert, and novel active genes (Fan et al., 2020). Orthologs are genes in different genomes that originated from the splitting of taxonomic lineages, whereas paralogs are genes in the same genome that originated from gene duplication (Thornton and DeSalle, 2000; Song et al., 2016). Paralogs typically have different functions, whilst orthologs may have the same function (Tatusov et al., 1997; Song et al., 2016). Our current comparative and phylogenetic analysis revealed 13 pairs of putative orthologous proteins between *Z. jujuba* (ZjMYBs) and *Z. mauritiana* (ZmMYBs). In contrast, 10 pairs of paralogous MYB family proteins were identified in *Z. jujuba.* Similarly, 2 pairs of paralogous MYB family proteins were also identified in *Z. mauritiana* (**Figure 1**). MEME was used to identify conserved motifs in both species’ MYB proteins.

A number of flavonoid-related MYB TFs have already been discovered in plants that regulate secondary metabolism in the plant species (Chezem et al., 2016; Wang et al., 2017). For example, the TT2, the first PA-related MYB TF discovered, activates *DFR*, *ANS*, and *ANR*, causing proanthocyanidin biosynthesis in the seed coats of *A. thaliana* (Nesi et al., 2001). The unique regulators (VvMYBA1 and VvMYBA2), activate the *UFGT* genes of the anthocyanin pathway in grapevines (Kobayashi et al., 2002). Moreover, in grapevines, *VvMYBPA2* was discovered to be a direct regulator of numerous structural flavonoid pathway genes (Terrier et al., 2009). The MdMYB10 is an important regulator during apple fruit coloring (Espley et al., 2007). In apple fruits, MdMYB3 triggers flavonoid biosynthesis-related genes such as *CHI, CHS*, *FLS*, and *UFGT* (Wang et al., 2017). Similarly, the FaMYB10 controls anthocyanin pathway-related genes in strawberries, such as the majority of EBGs and LBGs in matured fruit receptacles during ripening (Medina-Puche et al., 2014). The MYB genes found in *H. brasiliensis*, *HbMYB1* overexpression in tobacco reduces stress-induced cell death (Peng et al., 2011; Wang et al., 2017). Furthermore, *PeMYB2, PeMYB11*, and *PeMYB12* have all been found to be involved in the development of red color in *Phalaenopsis* spp. flowers (Hsu et al., 2015).

The flavonoids like anthocyanin structural genes control plant pigmentation both temporally and spatially. The transcriptional level is primarily responsible for the regulation of structural genes (Li et al., 2017; Li et al., 2017a). R2R3-MYBs regulate anthocyanin transcription by influencing the transcripts of anthocyanin structural genes. Numerous anthocyanin R2R3-MYB TFs in various fruit plants have been isolated and categorized (Feng et al., 2015). A homolog of *PyMYB10*, *PyMYB10.1*, was cloned from ‘Aoguan’ (Feng et al., 2015). The transcriptome analysis on ‘Hongyang’ kiwifruit, revealed 9 R2R3 MYBs to be involved in anthocyanin synthesis during fruit development (Li et al., 2015). The *MYB5* and *MYBA* have been shown to be positive potent inducers in the early stages of development of ‘Hongyang’ fruit (7 days after anthesis, DAA), where kiwifruit undergo a temporary anthocyanin formation, as well as later during fruit ripening (Li et al., 2015; Li et al., 2017; Li et al., 2017a). Some findings suggested that *VvMYB5a* and *VvMYB5b* in grapes, as well as *MdMYBA* in apples, could be stimulated by low temperatures, which then stimulated the expression of *ANS*, resulting in flavonoids like anthocyanin formation in fruit (Li et al., 2017).

The recognition of key MYB TFs associated with flavonoid accumulation during the period of fruit coloration in various species has greatly improved our understanding of the control mechanism of flavonoid biosynthesis in fruits. Based on our present transcriptome expression analysis four MYB genes (*ZmMYB*/*ZjMYB13*, *ZmMYB*/*ZjMYB44*, *ZmMYB*/*ZjMYB50*, and *ZmMYB*/*ZjMYB56*), were dramatically down-regulated or with low expression in *Z. mauritiana* green color fruit cultivar, which is probably concomitant with the lack of flavonoid color pigments content in green fruit cultivars of *Z. mauritiana*. For example, the *ZmMYB44* was significantly downregulated in *Z. mauritiana* fruit developmental stages while this gene (*ZjMYB44*) was upregulated in fruit developmental stages of *Z. jujuba* as shown in **Figure 4**. Furthermore, the expression of this gene was high in the junior fruit (JF) developmental stage in *Z. mauritiana* (**Figure 4**) while the highest expression was found in the full ripening fruit (RF) stage in *Z. jujuba* (**Figure 4**). Similarly, the high expression of *ZmMYB56* was found in JF (Junior fruit) and BWRF (Before white ripening fruit) stages and then decreased the expression in WRF (White ripening fruit), HRF (Half ripening fruit), and RF (Ripe fruit) stages of fruit development in *Z. mauritiana*. Further, the expression of *ZmMYB*/*ZjMYB13* and *ZmMYB*/*ZjMYB50* genes were downregulated in *Z. mauritiana* fruit developmental stages and was upregulated in *Z. jujuba* fruit as presented earlier in **Figure 4**. Moreover, the transient overexpression of *AcMYB5-1/5-2/A1-1* in *N. benthamiana* leaves increased expression patterns of *NtANS* and *NtDFR*, suggesting that those MYB TFs may be involved in the flavonoid synthesis pathway (Li et al., 2017). Furthermore, the total flavonoid content of jujube decreased as the fruit ripened (Wu et al., 2012), indicating the low expression of corresponding genes. Since the transient expression assay in *Z. jujuba* fruit, *ZjMYB44* significantly increased flavonoid accumulation indicating that this gene can stimulate the flavonoid content accumulation in the jujube fruit as described in **Figure 6A**.

## 5 Conclusion

In this study, we conducted the first transcriptome-wide analysis of *Z. mauritiana* and *Z. jujuba MYB* superfamily genes, which included 56 ZmMYB and 60 ZjMYB transcription factors (TFs) genes, in *Z. mauritiana* and *Z. jujuba*, respectively. The phylogenetic analysis revealed 13 pairs of putative orthologous proteins between *Z. jujuba* (ZjMYBs) and *Z. mauritiana* (ZmMYBs). In contrast, 10 pairs of paralogous MYB family proteins were identified in *Z. jujuba.* Similarly, 2 pairs of paralogous MYB family proteins were identified in *Z. mauritiana*. Four similar MYB genes (*ZmMYB/ZjMYB13, ZmMYB/ZjMYB44, ZmMYB/ZjMYB50*, and *ZmMYB/ZjMYB56)* from *Z. mauritiana* and *Z. jujuba* were identified as candidate key regulating flavonoid biosynthesis genes through transcriptomic expression analysis. Moreover, *in vivo* transient expression of *ZjMYB44* significantly increased flavonoid accumulation around the injection site, and the *ZjMYB44* gene was also transiently highly expressed in fruit, indicating that this gene can influence flavonoid content and lead to coloration in *Ziziphus* fruit. Our research results provide an important understanding of the molecular mechanism of flavonoid biosynthesis resulting in fruit coloration in *Ziziphus*, laying the groundwork for further functional characterization and genetic improvement of *Ziziphus* (*Z. mauritiana* and *Z. jujuba*) fruit coloration.

## Data availability statement

The original contributions presented in the study are included in the article/**Supplementary Material**. Further inquiries can be directed to the corresponding authors.

## Author contributions

NM wrote this article, NM and ZLuo did the laboratory research work. XZ helped in the laboratory research work. MY, ZLiu, and ML critically revised this manuscript. All authors contributed to the article and approved the submitted version.
