# Supplementary material for: Transcriptome-wide expression analysis of MYB gene family leads to functional characterization of flavonoid biosynthesis in fruit coloration of Ziziphus Mill
Source: Front Plant Sci. 2023 May 12;14:1171288. doi: 10.3389/fpls.2023.1171288 (PMC10213540; doi:10.3389/fpls.2023.1171288)
Supplement: Supplementary file 1 [file DataSheet_1.docx]

Supplementary Material

**Transcriptome-wide expression analysis of *MYB* gene family leads to functional characterization of flavonoid biosynthesis in fruit coloration of *Ziziphus* Mill.**

**Noor Muhammad ^1, 2,^** **^†^ , Zhi Luo ^1, 2,^** **^†^, Xin Zhao^1, 2^, Meng Yang^1, 2^, Zhiguo Liu^1, 2^,* and Mengjun Liu ^1, 2^,***

^1^ College of Horticulture, Hebei Agricultural University, Baoding 071001, China

^2^ Research Center of Chinese Jujube, College of Horticulture, Hebei Agricultural University, Baoding 071001, China

# Supplementary Data

| **Table S1.** Designed primer sequences of DEGs for qRT-PCR expression analysis | | |
| --- | --- | --- |
|  | Sequence (5'->3') |  |
| *ZjMYB44-F* | GACAATCGCTCGCCTCCTTA | |
| *ZjMYB44-R* | GCCGTGAAGGTTACAGTCGT | |
| *ZjMYB50-F* | TTGGCGCAAACATTCATAATAAAAT | |
| *ZjMYB50-R* | TGTTCGTCCCGGTAACTGTG | |
| *ZjMYB56-F* | TGGTCACTAATTGCTGGGCG | |
| *ZjMYB56-R* | TGTTGGTTTGAGTCGATGGGT | |
| *ZjMYB13-F* | TTGTTGTTGGAGTCATAGCCA | |
| *ZjMYB13-R* | TCTGAAGTAAAGAAGGAACATCCG | |
| *ZmMYB44-F* | AATCGATTCCTGGTCGCTCC | |
| *ZmMYB44-R* | TAAGGAGGCGAGCGATTGTC | |
| *ZmMYB50-F* | TTGGCGCAAACATTCATAATAAAAT | |
| *ZmMYB50-R* | TGTTCGTCCCGGTAACTGTG | |
| *ZmMYB56-F* | AGGTCTAATGAGGGGTGGGAA | |
| *ZmMYB56-R* | GAAGCCGCCCAGCAATTAGT | |
| *ZmMYB13-F* | TTGTTGTTGGAGTCATAGCCA | |
| *ZmMYB13-R* | TCTGAAGTAAAGAAGGAACATCCG | |
| ZjMYB primers for transient expression | | |
| *ZjMYB44-F* | GCGGCGTTCTACCTCTTGTA | |
| *ZjMYB44-R* | GGTTGGAGTGGTAGCCGTC | |
| *ZjMYB50-F* | ATCATAGAGGCGACGGTG | |
| *ZjMYB50-R* | TGGTGGAGTTGGAGCAGT | |
| *ZjMYB56-F* | TCCGCCGACTATGTAATCC | |
| *ZjMYB56-R* | TTTTCTTGTTCCTCCCTCA | |
| *ZjMYB13-F* | CAGTTCTTCCGTTTGTTTCA | |
| *ZjMYB13-R* | ATTATCATTTCCATTGCCAT | |
| Vector primers and reference primers | | |
| pCambia1302-GFP-F:TGGAGAGGGTGAAGGTGA | | |
| pCambia1302-GFP-R:CTTGAAGAAGTCGTGCCG | | |
| ZjACT-F AGCCTTCCTGCCAACGAGT | |  |
| ZjACT-R TTGCTTCTCACCCTTGATGC | |  |

# Supplementary Figures


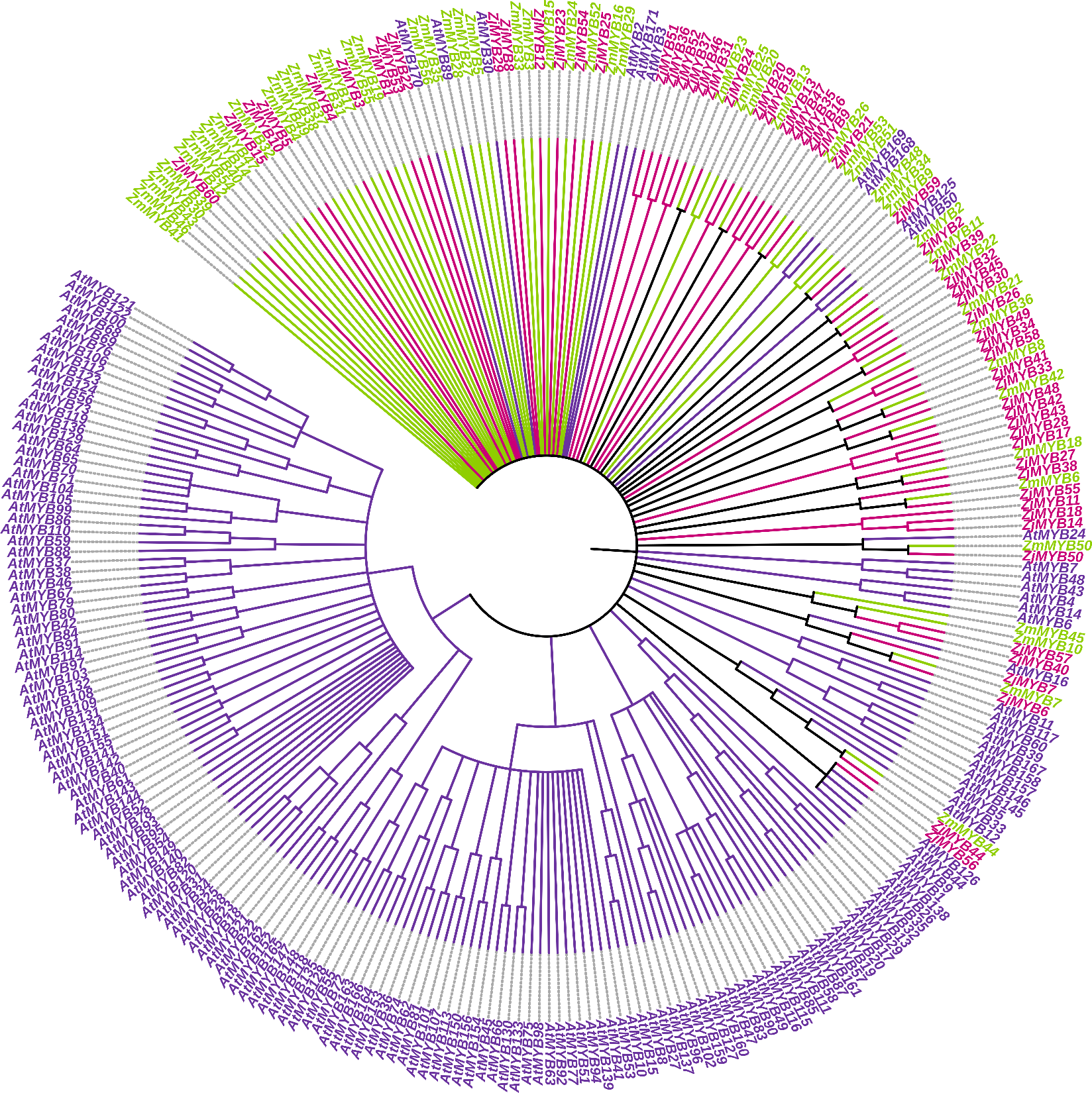


**Figure S1.** Comparative phylogenetic analysis of *Z. mauritiana*, *Z. jujuba*, and *A. thaliana* MYB TFs represented by different colors. The AtMYB represents the *A. thaliana* MYB TFs, while the ZmMYB and ZjMYB represent *Z.mauritiana* and *Z. jujuba* MYB TFs respectively. Further MYBs of *A. thaliana*, *Z.mauritiana*, and *Z. jujuba,* are labeled with light blue, yellow-green, and red-violet colors respectively.


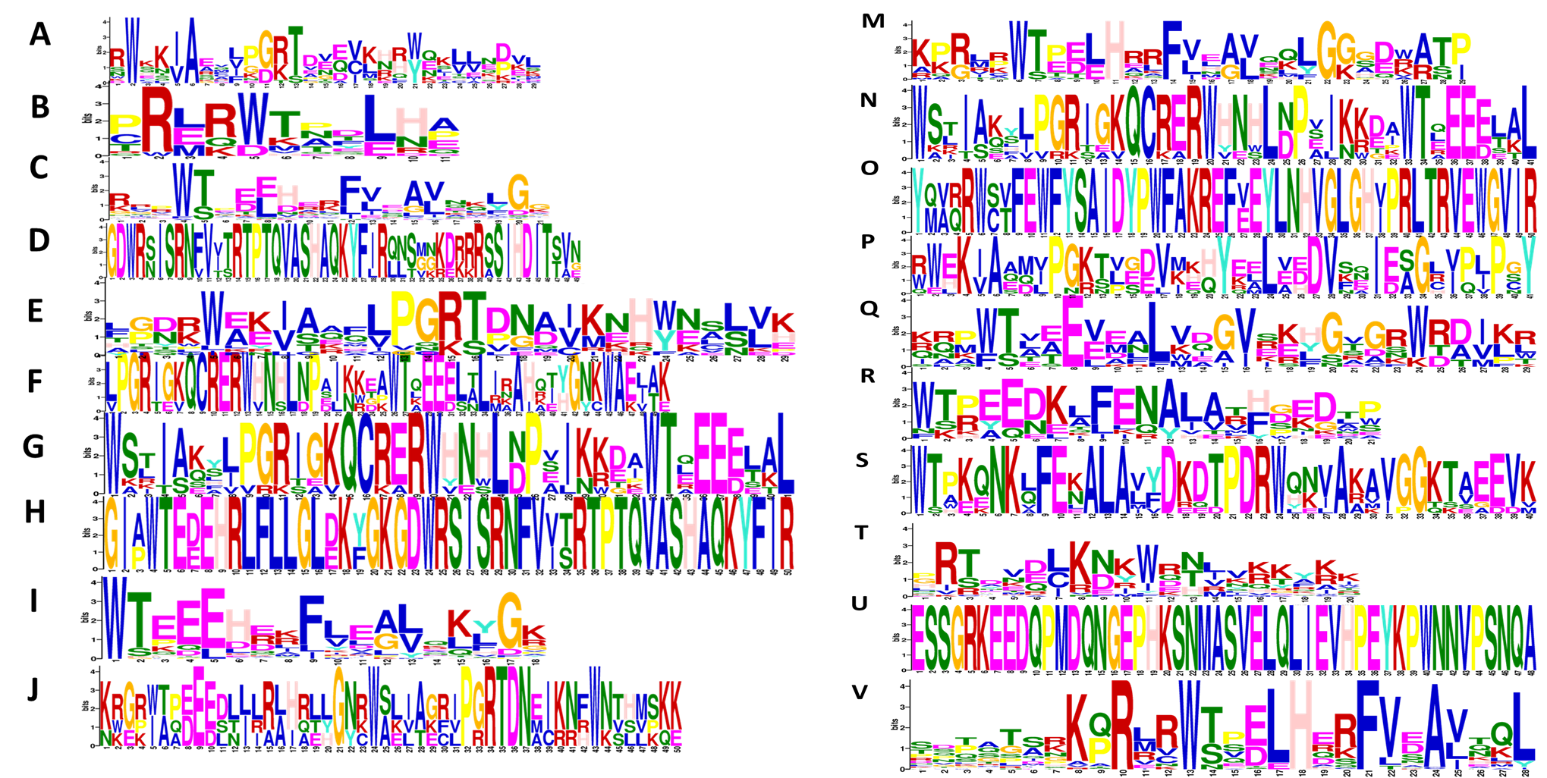


**Figure S2**. Sequence logos of the MYB repeats of *Z. mauritiana* (Zm), and *Z. jujuba* (Zj). A to J & M to V respectively. A to J indicates the MYB protein logos based on multiple alignment analyses of 56 ZmMYB proteins. M to V designates the MYB protein logos of 60 ZjMYB proteins.

**
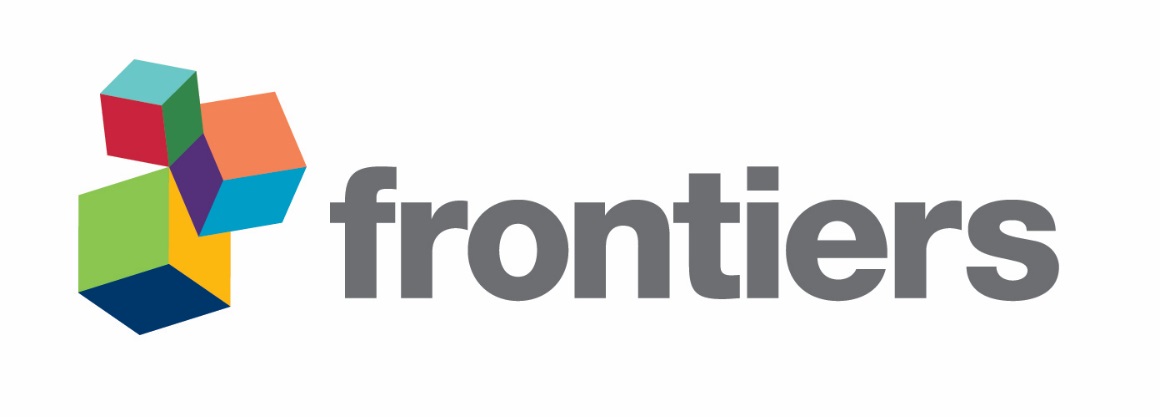
**
